# Cohort profile: the Swedish Evaluation Through Follow-up Study of Learning Later in Life (UGU-LIFE)

**DOI:** 10.1136/bmjopen-2026-125059

**Published:** 2026-10-21

**Authors:** Isabelle Hansson, Anne Ingeborg Berg, Pär Bjälkebring, Sandra Buratti, Jonas Burén, Linda Hassing, Anna-Carin Jonsson, Lina Jonsson, M Lindwall, Andreas Segerberg, Valgeir Thorvaldsson, Mikael Landén, Alli Klapp, Martin Lövdén

**Affiliations:** 1 Department of Psychology, University of Gothenburg, Gothenburg, Sweden; 2 Department of Surgery, University of Gothenburg, Gothenburg, Sweden; 3 Department of Education and Special Education, University of Gothenburg, Gothenburg, Sweden; 4 Department of Psychiatry and Neurochemistry, Institute of Neuroscience and Physiology at the Sahlgrenska Academy, University of Gothenburg, Gothenburg, Västra Götaland, Sweden; 5 Department of Medical Epidemiology and Biostatistics, Karolinska Institutet, Stockholm, Sweden

**Keywords:** Aging, Cognition, Longitudinal studies, Health, REGISTRIES, GENETICS

## Abstract

**Abstract:**

**Purpose:**

The Swedish Evaluation Through Follow-up Study of Learning Later in Life (UGU-LIFE) was established to study the factors that shape lifelong learning and advance evidence-based means to facilitate learning in older age.

**Participants:**

UGU-LIFE builds on the Evaluation Through Follow-up (*Utvärdering Genom Uppföljning*, UGU, in Swedish) study, which consists of nationally representative samples of Swedish birth cohorts. The two oldest cohorts, born 1948 (n=11 945) and 1953 (n=9927), were assessed at age 13 years and invited for follow-up assessments as part of UGU-LIFE in 2025 (age 72/77 years; n=5738).

**Findings to date:**

Data collection in childhood included a survey (on school and family conditions), cognitive tests and school administrative data. The follow-up assessment in late adulthood included a survey (on personal and contextual factors), cognitive tests, learning tasks and saliva sampling for DNA extraction. In addition, registry data were collected from Statistics Sweden (census), the National Board of Health and Welfare (medical records) and the Swedish National Archives (military conscription). Analysis of selectivity at follow-up showed higher retention rates among individuals with higher educational level and better cognitive ability in childhood, which was only partially explained by selectivity in survival.

**Future plans:**

Data collected in UGU-LIFE will be used to describe the predictors of lifelong learning, the factors that influence learning gains and engagement in learning in older age and the mechanistic pathways through which these factors affect learning in older age. Work to add birth records and geocoding to the data is ongoing. A subsample of participants will be invited to take part in an in-depth data collection of learning an ecologically relevant task over several days. The findings will be used to design and test interventions aiming to facilitate learning in older age.

**STRENGTHS AND LIMITATIONS OF THIS STUDY**

- The Swedish Evaluation Through Follow-up Study of Learning Later in Life includes population representative samples of individuals born in 1948 and 1953 (n=21 872).
- Participants were followed over six decades to assess how factors in childhood predict learning in older age.
- Cognitive testing in both childhood and late adulthood enables investigation of lifespan changes in cognitive functioning.
- DNA sampling and comprehensive nationwide registry linkage provide information on genetic, health and socioeconomic factors across the lifespan.
- A limitation is the low retention rate at follow-up (26.2% of the original cohort), with selective participation particularly related to childhood cognitive ability and educational level.

## Introduction

 The knowledge and skills required for functioning in a society change during the life course due to both societal changes (eg, technological advancements)^1^ and individual life transitions (eg, retirement).^2^ In adult life, acquiring new knowledge and skills is essential for personal and professional development. In older age, learning is fundamental for maintaining functional independence.^3 4^ Much of this learning occurs as a part of daily life and is important, for example, in reasoning about medical decisions, identifying misinformation on social media and avoiding online scams. Learning may even be required to perform mundane tasks such as buying bus tickets, ordering food in a restaurant application, accessing medical records and booking appointments with healthcare providers.

Learning is a system’s processing of information from its environment, resulting in a change of system properties that alter its behavioural potential and reactions to this information.^5^ It is distinct from performance but can only be observed by changes in behavioural performance, which give us the critical measure of learning gain.^6^ Plasticity, the ability to learn, is theoretically separate from other cognitive abilities (eg, working memory), but these other abilities may also influence learning gains.^7–9^

Older adults face unique learning challenges, such as changing health, resources, cognitive functions, motivation and sociocultural and individual expectations.^10–13^ Differences among older adults are, however, best understood in a lifespan perspective, as the knowledge, skills and expectations acquired in childhood and throughout adulthood set the stage for later learning.^14–17^ While much focus has been put into understanding learning in the context of formal education in younger age,^18–20^ scientific knowledge about the unique challenges that older adults face in informal learning situations, the factors that explain individual differences among older adults in the ability to learn new skills, and the mechanisms that support learning later in life remain scarce. Societies are therefore generally unprepared for supporting their citizens’ lifelong learning. This lack of support negatively affects both societal and individual development.

The Swedish Evaluation Through Follow-up Study of Learning Later in Life (UGU-LIFE) was established to investigate the conditions shaping lifelong learning. By comprehensively charting the factors that explain individual differences in learning among older adults, the cohort provides a foundation for developing evidence-based means to facilitate learning throughout life, particularly for those who need it most. UGU-LIFE builds on the Evaluation Through Follow-up (*Utvärdering Genom Uppföljning*, UGU, in Swedish) study, a longitudinal research programme designed to evaluate education and career development in Sweden.^21^ UGU was initiated in 1961 by Statistics Sweden and the National Board of Education and includes nationally representative samples from 12 birth cohorts in Sweden. The two oldest birth cohorts, born in 1948 and 1953, were included in UGU when they were 13 years old (in sixth grade) in 1961 or 1966. In UGU-LIFE, the participants in these cohorts were reinvited for follow-up assessments in late adulthood. In this paper, we describe the baseline data collections in childhood and the first phase of follow-up assessments at age 72 (cohort 1953) or 77 (cohort 1948) years. This data collection establishes the empirical foundation for UGU-LIFE, enabling future planned studies to broaden the assessment of learning through complementary tasks, methodologies and targeted interventions.

### Cohort description

The two UGU cohorts included in UGU-LIFE were sampled based on date of birth (individuals born on the 5th, 15th or 25th of each month), corresponding to approximately 10% of the Swedish population in each birth cohort (see figure 1). The sampling frame was estimated at 12 166 individuals in the 1948 cohort and 10 723 individuals in the 1953 cohort. Data collection for the 1948 cohort was conducted between 8 and 27 May 1961. For the 1953 cohort, data collection took place between 9 and 28 May 1966. The data collections were conducted by Statistics Sweden in collaboration with the Department of Education at the University of Gothenburg.^21^ The students completed a survey and cognitive tests at school under the supervision of their teachers. School administrative data were collected by Statistics Sweden from year 6 until the end of compulsory school (year 9). A total of 11 950 individuals born in 1948 (98% of sampling frame) and 9927 individuals born in 1953 (93% of sampling frame) took part in the study. However, data for five individuals in the 1948 cohort could not be linked to the registries, reducing the linked dataset to 11 945 individuals.

**Figure 1:**
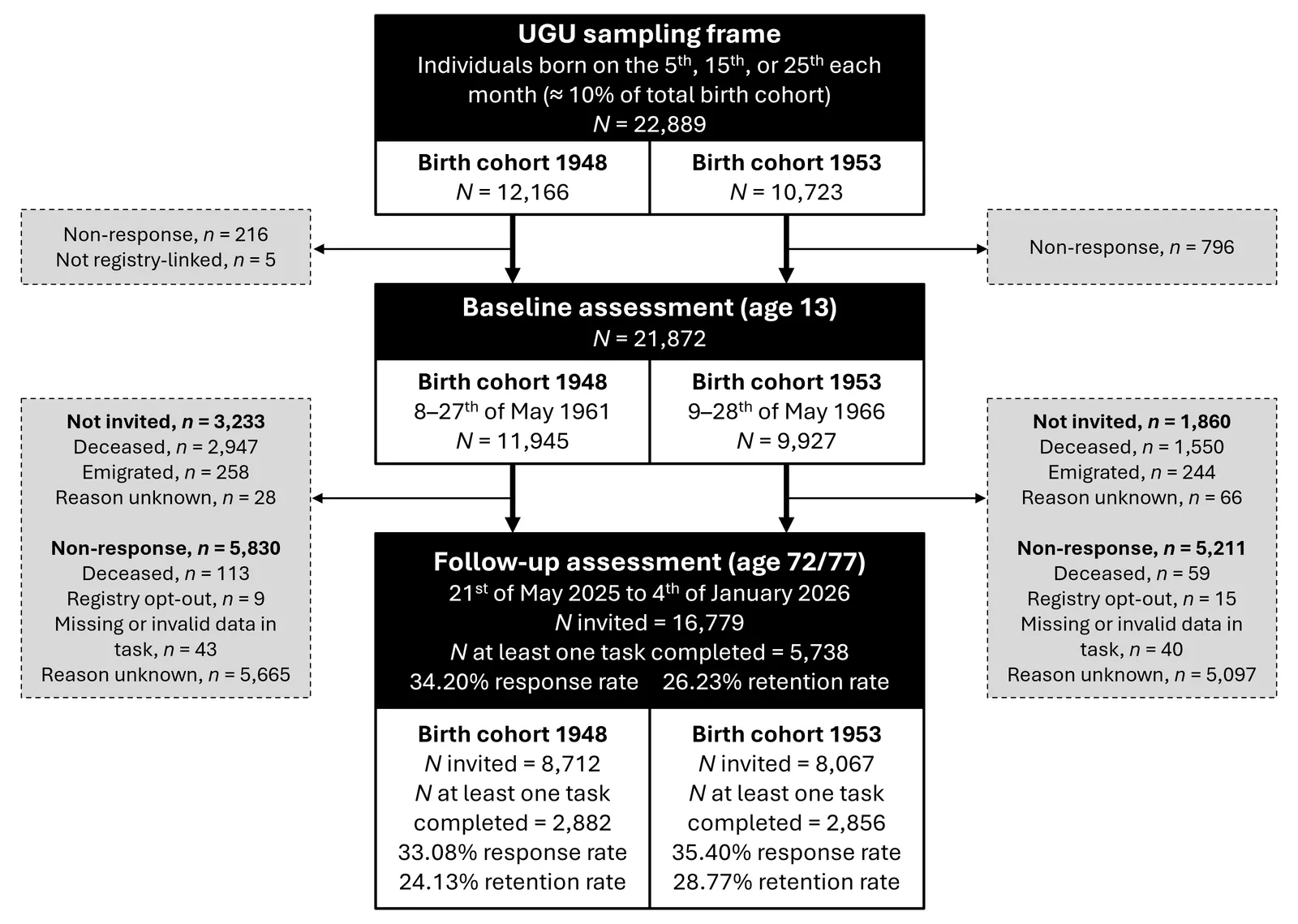
Flow chart of data collections in the Swedish Evaluation Through Follow-up Study of Learning Later in Life (UGU-LIFE). Registry linkage at follow-up was performed for all baseline respondents (n=21 872), apart from 24 individuals requesting registry opt-out.

Six decades later, we invited the participants in the two cohorts for follow-up assessments as part of UGU-LIFE. The data collection took place between 21 May 2025 and 4 January 2026, when participants were 72 or 77 years old. In collaboration with Statistics Sweden, we identified individuals who were alive and living in Sweden by the end of March 2025 as eligible for follow-up (n=16 779). Invitation letters, including a link to an online participation portal, were distributed digitally (when available; n=8889) or by post (n=7890) by Institutet för kvalitetsindikatorer (Indikator). Respondents completed a survey, cognitive tests, learning tasks and indicated interest in donating saliva for DNA extraction. Registry data was collected from Statistics Sweden, the National Board of Health and Welfare and the National Archives. Three reminders (16 June, 4 July and 2 August) were sent by post. The second reminder included a paper version of the survey, and the data collection portal was open until 4 January 2026. Volunteers for saliva sampling received a separate invitation letter together with a self-administered sampling kit, distributed between 23 June and 17 December 2025. Data collection for DNA extraction is ongoing and will be completed during autumn 2026 (the current paper includes samples collected between 27 June 2025 and 12 May 2026).

Participants received written information about the study and provided informed consent in the study portal before taking part (return of the paper questionnaire was considered consent). Saliva donation required separate written consent for genetic analysis. Registry linkage was granted for all members of the original UGU cohorts and was not conditional on participation in the follow-up. All living invitees were informed about the linkage and could opt-out by email, telephone or post (24 individuals did so).

For the purpose of describing follow-up participation, individuals were classified as having completed the follow-up when they provided the minimum amount of usable data required to distinguish meaningful participation from an effectively uncompleted record in at least one (out of seven) tasks. Using this definition, a total of 5738 (2882 from the 1948 cohort and 2856 from the 1953 cohort) individuals completed the follow-up assessment, resulting in a retention rate of 26% and a response rate among those invited of 34%. The response rate was lower than generally observed in well-established national surveys (eg, the SOM survey with a response rate around 50%)^22^ but comparable to similar studies on older adults in Sweden.^23^ In the following sections, the data collections are described in more detail.

### Baseline childhood assessment (age 13 years)

The data collected as part of the childhood assessment consist of survey responses, cognitive test scores and school administrative data via forms from Statistics Sweden. The questionnaire form filled in by the students in the 1948 cohort contained about 70 questions divided into four parts: leisure activities, plans, school and interests. The leisure activities formed six scales covering outdoor, verbal, technical, domestic, social and clerical areas. The school-related questions formed five scales: contact between children and parents, family’s attitude towards higher education, pupil’s anxiety in school situations, student’s interest in schoolwork and student’s peer contacts. The questionnaire form filled in by the students in the 1953 cohort contained about 70 questions divided into four parts: leisure activities, plans, school and occupations. The leisure activities formed five scales covering outdoor, verbal, technical, domestic and clerical areas. The school-related questions formed three scales: family’s attitude towards higher education, pupil’s anxiety in school situations and pupil adjustment to school.

The cognitive tests were identical for both cohorts and were designed to measure verbal knowledge (opposites), inductive reasoning (number series) and spatial ability (metal folding). All tests used a multiple-choice format and increased in difficulty across items. The opposites test consisted of 40 items in which participants selected the antonym of a target word from four response options. The number series test consisted of 40 items in which participants identified the next two numbers in a sequence based on six preceding numbers. The metal folding test consisted of 40 items in which participants viewed a two-dimensional pattern and selected, from four alternatives, the three-dimensional figure that would result if the pattern were folded.

School administrative data have been gathered for both cohorts in yearly updates until the participants left school. These data include information on the participants concerning demographics and school and municipality characteristics, including school form, class type, class structure, class character, grade, school choice up through the education system, special tuition, special teaching group, reduced courses, moved from the class and drop-out of compulsory school, mother’s and father’s education and occupation, birth month and sex. Academic achievement outcomes, including standardised knowledge tests and subject grades, have also been collected. A detailed description of data collected as part of the baseline assessment is presented in online supplemental material table 1.

### Follow-up assessment in late adulthood (age 72/77 years)

The data collected as part of the assessment in late adulthood consists of survey responses, cognitive test scores, learning performance, saliva sampling and registry data on health and socioeconomic conditions. Figure 2 shows an overview of the number of respondents to each task and task combination (intersection). Figure 3 shows an overview of the lifespan data coverage in the two birth cohorts.

**Figure 2:**
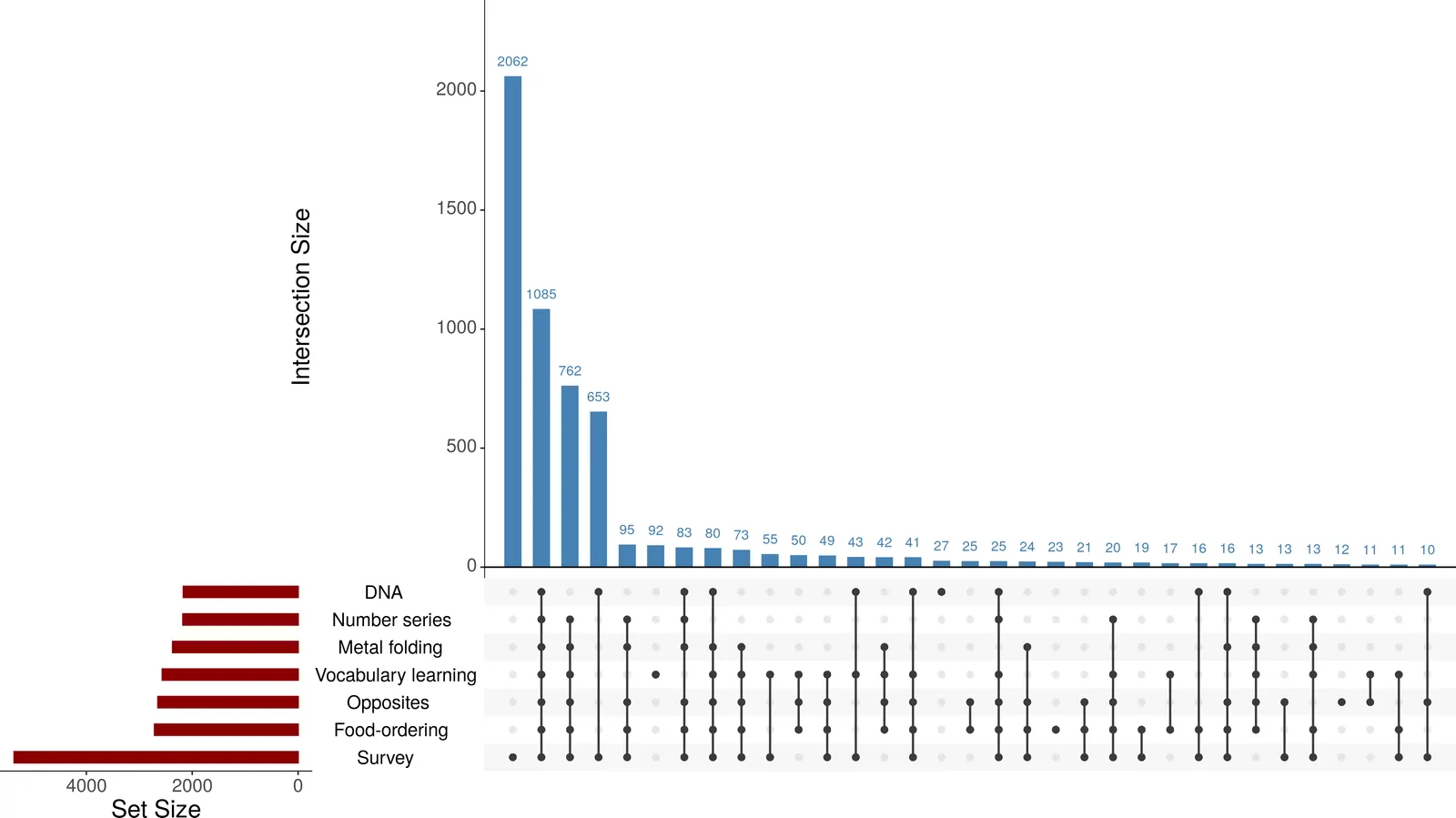
UpSet plot of participant frequency by task (set size) and task intersections (intersection size) in the follow-up assessment at age 72/77. For visualisation purposes, only intersections (ie, task combinations) with 10 or more observations (33 intersections) are included (in total 78 different task intersections were observed)*.* UGU-LIFE, the Swedish Evaluation Through Follow-up Study of Learning Later in Life.

**Figure 3:**
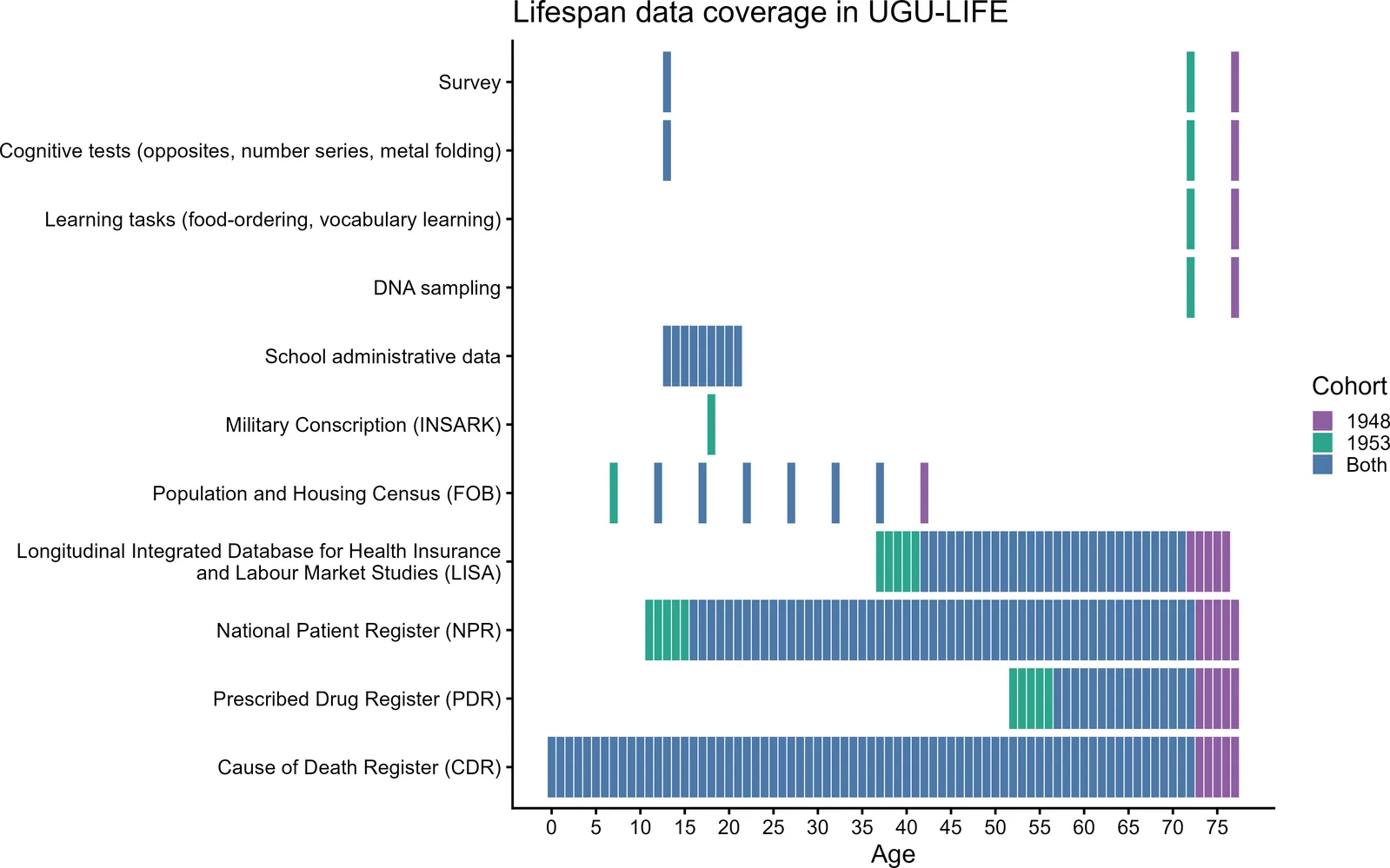
Overview of lifespan data coverage in the Evaluation Through Follow-up Study of Learning Later in Life (UGU-LIFE). Survey and cognitive tests at age 13 years and school administrative data were collected as part of the original UGU study. Survey and cognitive tests at age 72/77 years, learning tasks, DNA sampling and linkage to registry data from Statistics Sweden (FOB, LISA), the National Board of Health and Welfare (NPR, PDR, CDR) and the Swedish National Archives (INSARK; men only) were included in the UGU-LIFE follow-up. NPR has partial (regional) inpatient care from 1964 (age 11/16), full cover in psychiatric care from 1973 (age 20/25) and national coverage from 1987 (age 34/39). Outpatient care is available from 1997 (age 44/49) with full coverage from 2001 (age 48/53).

The questionnaire contained 56 questions with a total of 300 items. These were divided into seven parts: environmental context, learning engagement, motivation, expectations, personality and metacognition, resources and health. Questions on environmental context covered family and household conditions, retrospective school assessments, leisure activities and occupational history. Questions on learning engagement included frequency of learning engagement within theoretical (verbal, numeric, logical) and practical (mechanic, digital, motoric) domains, an adapted version of the Broad Learning Adult Questionnaire (BLAQ)^24^ and internet use. Measures on motivation included the Achievement Motives Scale (AMS-R)^25^ and the satisfaction subscale of the Basic Psychological Need Satisfaction and Frustration Scale (BPNSFS).^26^ Questions on expectations included perceived competence within theoretical (verbal, numeric, logical) and practical (mechanic, digital, motoric) learning domains, the Achievement Emotions Questionnaire (AEQ-S),^27^ the Implicit Theories of Intelligence (ITI) scale^28^ and the Awareness of Age-Related Change scale (AARC-10-SF).^29^ Personality was measured with a short form of the International Personality Item Pool-Five-Factor Model scale (MINI-IPIP)^30^ and metacognition with a short version of the Metacognitive Awareness Inventory (MAI).^31^ Questions on resources included financial satisfaction, cash margin, the UCLA Loneliness Scale,^32^ the Multidimensional Scale of Perceived Social Support scale (MPSS)^33^ and the Perceived Ageism Questionnaire (PAQ).^34^ Finally, questions within the health domain included the Satisfaction With Life Scale (SWLS),^35^ the Patient Health Questionnaire for depression (PHQ-9)^36^ and the Short Form Health Survey instrument (SF-36).^37^

Cognitive performance was assessed using the same tests of verbal knowledge (opposites), inductive reasoning (number series) and spatial ability (metal folding) that had been administered in year 6 at school (age 13), although the follow-up assessments were conducted in an online format. To reduce the risk of ceiling effects, five additional items were added to the verbal knowledge test (at the end of the test). These differences in administration and test format should be considered when making direct comparisons of performance across the two assessment occasions. In addition, confidence judgements were added for 10 preselected items from each test (30 in total). After the completion of each test, participants were presented with their answer to the selected items and asked to rate how sure they are that they have come to the correct answer on a confidence scale from 0% to 100% with intervals of 20%.

Learning performance was assessed with two short learning tasks, one primarily measuring procedural learning and one primarily measuring declarative learning. Procedural learning was measured using a food-ordering task designed as a hierarchical–sequential semantic decision task. The task simulated a food-ordering application (‘UGU-Eats’) and required participants to navigate a structured menu to place specific orders. At the beginning of each trial, participants were presented with an item to be ordered as quickly as possible. To complete a trial, participants had to make eight correct decisions that followed a hierarchical menu structure. The task consisted of eight trials. Trials 1–4 involved the same learnt sequence (ordering a big mackerel burger). Trial 5 introduced a novel sequence (medium raspberry sorbet), followed by the learnt sequence in trial 6 (big mackerel burger). Trial 7 introduced another novel sequence (small apple juice), and trial 8 again used the learnt sequence (big mackerel burger). The measure of performance for each trial was the total time (in seconds) required to complete the order, measured from trial onset to successful completion. Declarative learning was measured with a vocabulary learning task consisting of three study–test trials. The task involved learning associations between foreign words and Swedish translations. The foreign words were drawn from Sindarin, an artificial language created by J R R Tolkien, to minimise prior familiarity. In each trial, participants were presented with a list of 10 Swedish–Sindarin word pairs. Following a study phase of 60 s, participants completed a forced-choice test. Each Sindarin word was presented individually together with 10 Swedish response alternatives. After the 10 responses had been provided, the next study–test trial began. Performance was measured through the number of correct answers per trial. Detailed psychometric evaluation of the learning tasks, including score distributions, floor and ceiling effects, reliability, validity and derivation of learning-performance measures, is ongoing and will be reported in a separate validation study.

Saliva samples for DNA extraction were collected using a self-administered, non-invasive saliva collection kit (Oragene, DNA Genotek) that was mailed to participants. Samples were returned to the Karolinska Institutet Biobank (KIBB), where DNA was extracted continuously. Genotyping was performed using a genome-wide genotyping array (Illumina Infinium Global Screening Array—Multiple Disease version 3) designed to capture common genetic variation across the genome.

Registry data was collected from Statistics Sweden, the National Board of Health and Welfare and the Swedish National Archives for all participants in the original UGU samples (n=21 872), except for individuals asking not to be included in registry linkage (opt-out offered as part of data collection; n=24). Data from Statistics Sweden include information on demographic and socioeconomic conditions (including family background, residence, education, work life, income) from the Longitudinal Integrated Database for Health Insurance and Labour Market Studies (LISA; 1990–) and Population and Housing Census (FOB; 1960–1990). Data from the National Board of Health and Welfare include medical information from the National Patient Register (NPR; partial cover from 1964, national cover from 1987), the National Prescribed Drug Register (PDR; 2005–), and the National Cause of Death Register (CDR; full coverage). Because diagnoses in the National Patient Register depend on contact with specialist healthcare and subsequent clinical recording, absence of a registered diagnosis does not necessarily indicate absence of the underlying condition. Finally, data from the National Archive include assessments of health and cognitive functioning from military conscription (INSARK; 1971). Registry data will be updated continuously as new waves of data become available via Statistics Sweden and the National Board of Health and Welfare. We also plan to collect birth record data from the regional archives. A detailed description of data collected as part of the follow-up assessment is presented in online supplemental material table 2.

The entire data collection procedure, excluding saliva sampling and register linkage, was piloted on older adults from the same birth cohorts (ie, 1948 and 1953) who were born on dates other than those used to define the UGU samples (ie, not on the 5th, 15th, or 25th of the month). Invitations were sent to 1000 individuals drawn from the population registry. To assess mode effects, half of the sample (n=500) received paper-and-pencil versions of the cognitive tests. A total of 270 individuals completed the pilot study between 31 January and 28 April 2025. The pilot was used to assess feasibility and inform minor adjustments before the main data collection. Detailed analyses of mode effects and the psychometric comparability of the cognitive tests are being conducted separately and will be reported in a dedicated validation study.

### Findings to date

Data from UGU has been used to examine cohort differences in childhood cognitive ability,^38^ effects of educational reforms^39^ and predictive effects of childhood conditions (eg, family and school environment, cognitive ability and academic achievement) on educational^40 41^ and health-related^42–44^ outcomes. A complete list of UGU publications is available at https://www.gu.se/en/evaluation-through-follow-up/publications. However, no paper has yet been published on data collected as part of UGU-LIFE. The purpose of this section is therefore to describe participation and selectivity at follow-up.

The UGU-LIFE cohort (n=21 872) includes 11 139 (51%) men and 10 733 (49%) women. In 2025, 16 779 individuals were identified as eligible (postal address in Sweden end of March) for follow-up, and 4669 individuals were deceased (registered in CDR by 26 October). Individuals born in 1948 had a higher mortality rate (26%) than those born in 1953 (16%). The response rate (relative invited) was somewhat higher in the 1953 cohort (35%) compared with the 1948 cohort (33%). The follow-up sample of 5738 individuals includes 2704 (47%) men and 3034 (53%) women. The proportion of women at follow-up was slightly higher in the 1953 cohort (55%) than in the 1948 cohort (51%). A detailed description of sample characteristics at baseline and follow-up is presented in online supplemental material table 3.

### Selectivity at follow-up

To assess selectivity at follow-up, we calculated standardised mean differences (SMD) between the baseline (n=21 872) sample and the follow-up (n=5738) sample (*M*_follow-up_–*M*_baseline_/*SD*_baseline_). This selectivity was further decomposed into a mortality-associated (*M*_survivors_–*M*_baseline_/*SD*_baseline_) and an experimental (*M*_follow-up_–*M*_invited_/*SD*_baseline_) component.^45^ In these calculations, survivors include all individuals not identified as deceased in CDR (n=17 203) while invited include all individuals identified as alive and living in Sweden (n=16 779). We thereby evaluate how much of the total selectivity that can be attributed to selectivity in survival (likelihood to be alive at follow-up) versus participation among those invited to follow-up (experimental selectivity). Variables included in these analyses were: birth cohort, sex, birth region and region of residence (metropolitan vs non-metropolitan), parental background (Swedish vs foreign), parents’ education, childhood cognitive ability (total points of verbal, spatial and inductive tests), education, marital status, income and health. Information on birth cohort, sex, parental education and childhood cognitive ability were gathered from the original UGU data. Data on birth region, parental background, education, marital status and income come from Statistics Sweden. Health was calculated as weighted score of diagnoses (Charlson Comorbidity Index)^46^ reported in the National Patient Register. For education, marital status and health, we selected one indicator from early midlife (1990, age 37/42) and one from late adulthood (latest available data source, 2023–2025 depending on registry). Measures in early midlife thereby enable assessment of mortality risk (91% of deaths occurred after 1990) while measures in late adulthood capture how current conditions (among those still alive) shape experimental selectivity. Variable coding, descriptive statistics and missingness patterns are presented in online supplemental material table 3, 4. Figure 4 shows an overview of the results, which are summarised below (all effect sizes are presented in online supplemental material table 5). For interpretation of dichotomous variables, effect sizes are supplemented with rate ratios (incidence rate group a/incidence rate group b) which are expressed as percentage differences in text.

**Figure 4:**
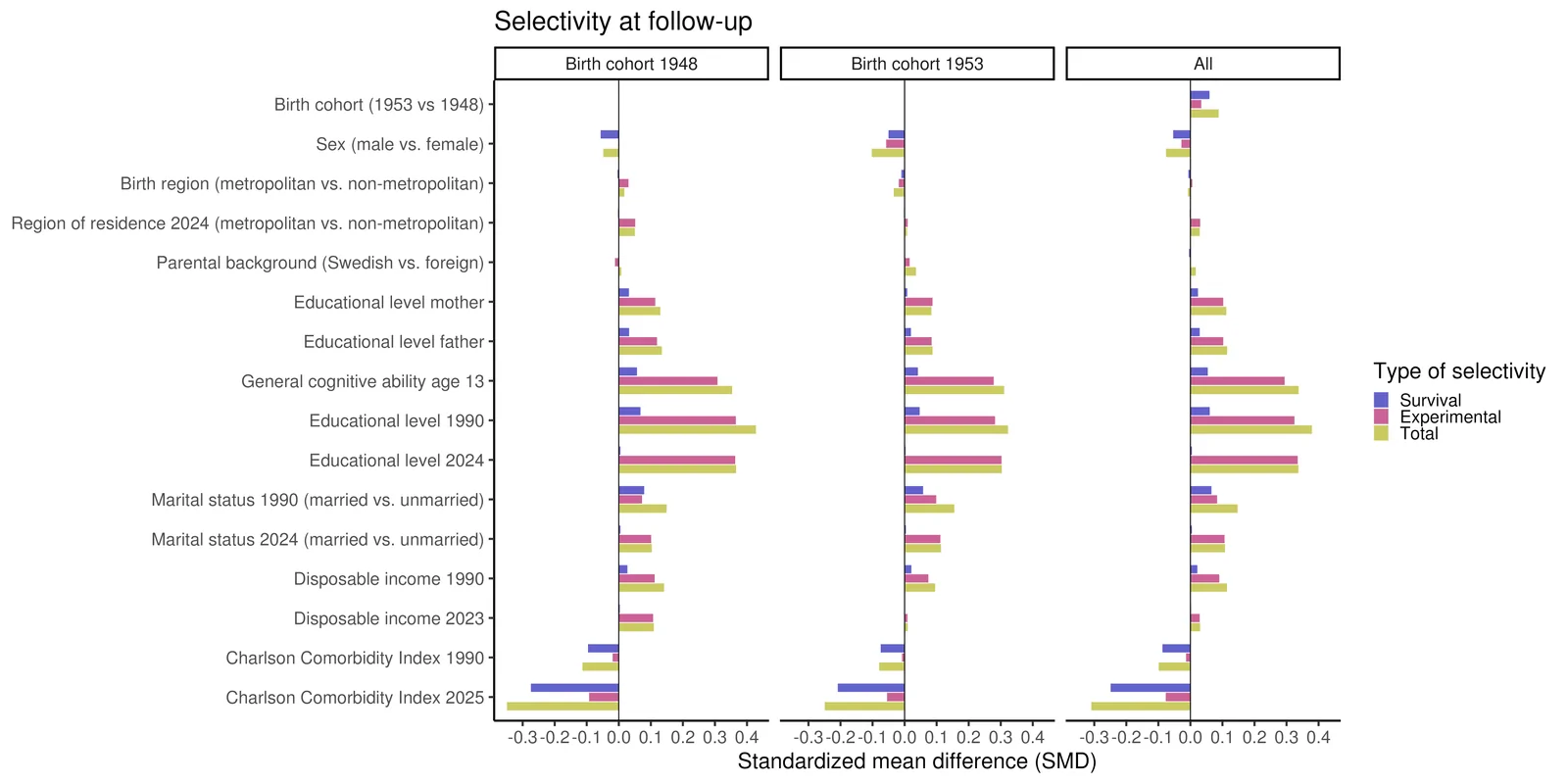
Selectivity (standardised mean difference) in survival (mortality-associated selectivity), follow-up (experimental selectivity) and retention (total selectivity) during the follow-up assessment at age 72/77 (2025).

Compared with those born in 1948, individuals born in 1953 were 13% more likely to be alive at follow-up and 7% more likely to complete the follow-up assessment (experimental selectivity). The mortality-associated component accounted for 68% of the total cohort-related selectivity at follow-up. Men were 10% less likely than women to be alive at follow-up and 5% less likely to complete the follow-up assessment. The mortality-associated component accounted for 71% of the total sex-related selectivity at follow-up.

Respondents at follow-up had higher parental education, higher childhood cognitive ability and higher educational level (*SMD*=0.11–0.38). Only a smaller proportion (16%–26%) of this selectivity could be attributed to selectivity in survival. Individuals married in 1990 were 39% more likely than unmarried to complete the follow-up assessment (total selectivity) and a large proportion (45%) of this selectivity could be attributed to selectivity in survival. Marital status in 2024 was also related to selectivity in follow-up, with married individuals being 25% more likely than unmarried to respond to the invitation. Higher income in 1990 was associated with a higher probability of participating in the follow-up (*SMD*=0.11; mortality-associated component=19%), but income in 2023 was only marginally related to response probability. Respondents at follow-up had better health in 1990 (*SMD*=−0.10), but most of this selectivity (89%) could be attributed to selectivity in survival. Among individuals alive at follow-up, respondents had slightly better health compared with non-responders. Finally, only marginal selectivity effects were found for birth region, region of residence and parental background.

### Selectivity in task completion at follow-up

Among the total follow-up sample of 5738 individuals, 93% (n=5363) completed the survey (n=2207 online, n=3156 via paper), 47% (n=2711) the food-ordering task, 46% (n=2647) the opposites test, 45% (n=2564) the vocabulary learning task, 41% (n=2369) the metal folding test, 38% (n=2175) the number series test and 38% (n=2166) donated saliva for DNA extraction. The average number of tasks completed per participant was 3.48 (*SD*=2.50). Task-specific overlap is presented in figure 2, cohort-specific frequencies are presented in online supplemental material table 6.

Selectivity in task completion was assessed by calculating SMD between each task-specific sample and the total follow-up sample (*M*_task_–*M*_follow-up_/*SD*_follow-up_). Variables included in these analyses were: birth cohort, sex, region of residence, childhood cognitive ability and the most recent registry data on education, marital status, income and health. Figure 5 shows an overview of the results, which are summarised below (all effect sizes are presented in online supplemental material table 7).

**Figure 5:**
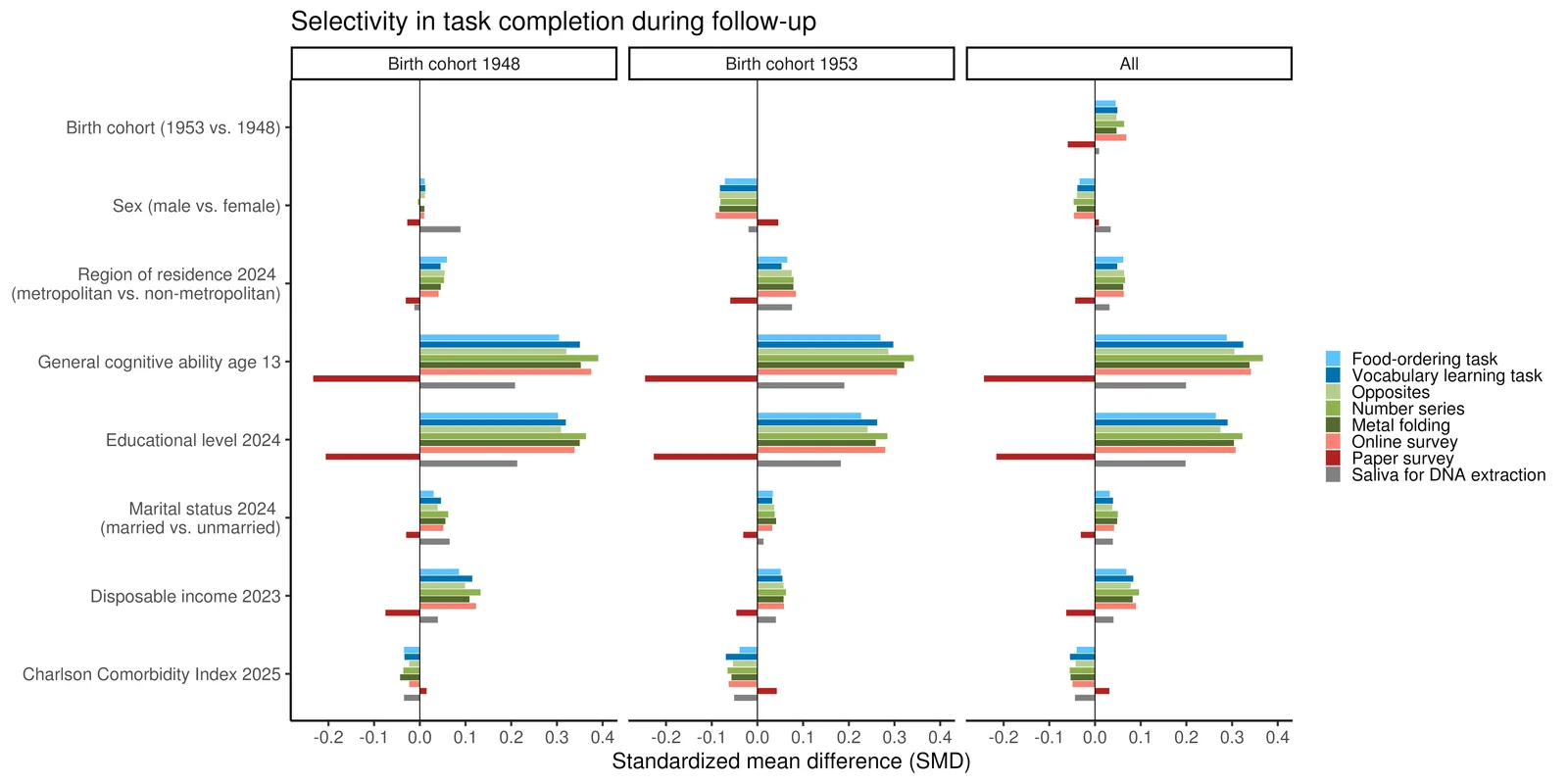
Selectivity (standardised mean difference) in task completion during the follow-up assessment at age 72/77 (2025).

Individuals born in 1953 were 10%–15% more likely than those born in 1948 to complete online (learning tasks, cognitive tests and online survey) tasks and 11% less likely to complete the paper survey, but no notable difference was found in the willingness to donate saliva for DNA extraction. Men were 7%–9% less likely than women to complete online tasks and 7% more likely to donate saliva for DNA extraction, while almost no sex differences were found in paper survey completion. Sex differences were primarily shown in 1953 cohort, where men were 13%–17% less likely to complete online tasks. For DNA sampling, however, the pattern was reversed, with larger sex differences in the 1948 cohort (men 20% more likely to donate saliva).

Participants completing online tasks had better childhood cognitive ability and higher educational level compared with the total follow-up sample (*SMD*=0.26–0.37), an effect largely driven by poorer cognitive ability and lower education among paper survey respondents. Online responders also had higher income, slightly better health and a larger proportion of married individuals and people residing in metropolitan areas, but the effect sizes were much weaker compared with those observed for cognitive ability and education. Participants who donated saliva had better cognitive ability and higher educational level (*SMD*=0.20), but only marginally higher income and better health compared with the total follow-up sample. Married individuals and people residing in metropolitan areas were slightly more likely to donate saliva.

### Strengths and limitations

UGU-LIFE is a rare population-based cohort that combines prospectively collected childhood data with a follow-up assessment six decades later, repeated cognitive testing across the lifespan, direct measures of learning in older age, genetic data and comprehensive nationwide register linkage. The cohort builds on nationally representative samples of individuals born in Sweden in 1948 and 1953 and provides a valuable opportunity to study how childhood conditions shape learning and related outcomes (eg, health) in older age. The long follow-up period allows for investigation of developmental pathways across most of the life course. Another strength is the breadth of available data. Information from surveys, cognitive tests, learning tasks, DNA sampling and nationwide registers can be combined within the same cohort. The register linkage provides detailed information on socioeconomic conditions, education, work life, health, medication and mortality, enabling analyses of factors that may influence learning across adulthood. In addition, the cognitive tests administered in late adulthood are based on the same measures that participants completed in childhood, a rare feature in cohort studies,^47 48^ allowing assessment of long-term continuity and change in cognitive functioning. However, direct comparisons of absolute performance across assessments require caution because administration conditions differed between childhood and late adulthood. The extent to which these differences influence score comparability is currently being evaluated in a separate psychometric validation study.

UGU-LIFE was specifically designed to study learning in older age. In contrast to many ageing studies that focus primarily on cognitive performance, the cohort data include direct measures of learning performance together with assessments of learning engagement, motivation, expectations, metacognition, resources and health. This combination provides opportunities to study both predictors of learning and mechanisms that may explain individual differences in learning gains among older adults.

A major limitation of UGU-LIFE is the low retention rate and selective attrition at follow-up. Loss to follow-up limits the representativeness of the sample relative to the original nationally representative cohorts. As expected after six decades, both mortality-associated and experimental sources of selectivity were observed. Individuals with higher educational level and cognitive ability were more likely to take part in the follow-up. These patterns indicate that findings based on the follow-up sample may underestimate outcomes associated with lower education or lower childhood cognitive ability. An important lesson is the value of offering multiple modes of participation when following older adults. Although completion of the online tasks was more common among individuals with higher educational level and cognitive ability, the option to respond using a paper questionnaire substantially increased participation and broadened coverage of the target population. Future studies aiming to assess learning and cognition in older age may benefit from combining digital and traditional modes of data collection. A further limitation concerns the use of healthcare registry data to characterise health. Because diagnoses in the National Patient Register are recorded in connection with healthcare contacts, observed morbidity may partly reflect healthcare-seeking behaviour, access to care, referral patterns and diagnostic practices, introducing a risk of consultation bias.

### Collaboration

UGU-LIFE welcomes collaboration with researchers whose proposed work is of high quality and consistent with the scientific aims of the study, the ethical approvals governing the study and applicable data protection legislation. The cohort data are particularly well suited for research studying the factors influencing learning and related factors in later life from a lifespan perspective.

The UGU-LIFE dataset is currently being assembled, quality controlled, documented and integrated with registry and genetic data. Work to collect additional data on subsamples and work to retrieve birth records are ongoing. Initial analyses focusing on the validation of questionnaires and tasks are currently being conducted by the research group. In parallel, work on establishing the data sharing framework for the complete dataset is ongoing. Individual-level UGU-LIFE data are therefore not yet available to external researchers. We expect a controlled-access data sharing framework of the entire dataset to be established during the coming years as data collection is completed and data quality assurance, documentation and governance procedures are finalised.

Once data access procedures have been finalised, researchers will be able to apply for access through a disclosure process administered by the University of Gothenburg (https://www.gu.se/en/evaluation-through-follow-up/disclosure-of-ugu-data). Data documentation and analytic codes will be published continuously via repositories on the Open Science Framework (https://osf.io/6mb3y) and GitHub (https://github.com/ugu-life).

## Supplementary material

10.1136/bmjopen-2026-125059 online supplemental file 1

## Data Availability

Data may be obtained from a third party and are not publicly available.
