# Supplementary material for: Cohort profile: the Swedish Evaluation Through Follow-up Study of Learning Later in Life (UGU-LIFE)
Source: BMJ Open. 2026 Oct 21;16(10):e125059. doi: 10.1136/bmjopen-2026-125059 (PMC13691816; doi:10.1136/bmjopen-2026-125059)
Supplement: Supplementary file 1 [file bmjopen-2026-125059.reviewer_comments.pdf]

PEER REVIEW HISTORY

BMJ Open publishes all reviews undertaken for accepted manuscripts. Reviewers are asked to complete a checklist review form and are provided with free text boxes to elaborate on their assessment. These free text comments are reproduced below.

ARTICLE DETAILS

Title (Provisional)

Cohort profile: The Swedish Evaluation Through Follow-up Study of Learning Later in Life (UGU-LIFE)

Authors

Hansson, Isabelle; Berg, Anne Ingeborg; Bjälkebring, Pär; Buratti, Sandra; Burén, Jonas; Hassing, Linda; Jonsson, Anna-Carin; Jonsson, Lina; Lindwall, M; Segerberg, Andreas; Thorvaldsson, Valgeir; Landén, Mikael; Klapp, Alli; Lövdén, Martin

---

VERSION 1 - REVIEW

**Reviewer**	**1**
**Name**	**Huizhen, Cao**
**Affiliation**	**Fuzhou University Affiliated Provincial Hospital**
**Date**	**21-Aug-2026**
**COI**	**None**

This paper aims to established to investigate the conditions shaping lifelong learning. The cohort reframing aging as a time of active knowledge production and inclusive lifelong learning systems and age-friendly societies. The greater impact of lifelong learning and selective attrition of the order people can be associated with activities of daily living, vision and hearing. Details of the cohort how to evaluate these factors are, however, not clear.

**Reviewer**	**2**
**Name**	**Richards, Marcus**
**Affiliation**	**University College London**
**Date**	**25-Aug-2026**
**COI**	**None**

Education though out life is an important yet a relatively under-studied topic. This is a well-written and comprehensive description of the UGU-LIFE study. However, I would suggest three minor revisions. First, re the Strengths subsection, while the simultaneous availability

of cognition in childhood and adulthood is rare, it is not - as claimed by the authors - unique. For example, both are available in the British 1946 birth cohort and follow-ups of the Scottish Mental Surveys. Second, while the authors comment on the low retention rate for follow-up (26.2%), this is in fact a serious limitation. Third, the authors should comment on the risk of consultation bias in the use of health registry data.

**Reviewer**	**3**
**Name**	**Caffo, Alessandro Oronzo**
**Affiliation**	**Universita degli Studi di Bari Aldo Moro**
**Date**	**25-Aug-2026**
**COI**	**None**

This manuscript presents the cohort profile of the Swedish Evaluation Through Follow-up Study of Learning Later in Life (UGU-LIFE), which builds on two nationally representative Swedish birth cohorts originally assessed at age 13 and followed up approximately six decades later. The study combines childhood survey and cognitive data with cognitive testing, direct learning tasks, psychosocial measures, nationwide registry data, and genetic sampling in later life.

Overall, this is a valuable and potentially important research resource. The exceptionally long follow-up period, the repeated administration of cognitive measures, and the integration of multiple data sources provide considerable opportunities to investigate learning and cognitive functioning from a lifespan perspective. The manuscript is generally well organised, and the explicit examination of mortality-related and experimental selectivity is particularly welcome.

I believe that the cohort profile is suitable for publication. However, several aspects of the reporting require clarification or correction before the manuscript can provide a sufficiently precise and reproducible description of the cohort. I therefore recommend major revision, although the issues identified below appear to be addressable without fundamental changes to the study.

Major comments

Inconsistency in the distribution of sex at follow-up

There is a clear inconsistency between the overall and cohort-specific figures reported in Table 3. In the overall follow-up sample, the table reports 3,034 men and 2,704 women. However, the cohort-specific figures sum to 2,704 men (1,406 + 1,298) and 3,034 women (1,476 + 1,558). The cohort-specific percentages also indicate that women represented approximately 51% of the 1948 cohort and 55% of the 1953 cohort at follow-up.

The same apparent inversion is reproduced in the main text, where the authors state that the follow-up sample included 3,034 men and 2,704 women. The authors should correct these figures and verify whether the error is confined to the presentation of the descriptive data or also affects the sex-related selectivity analyses and the corresponding figures.

Definition and calculation of selectivity indicators

The analysis of selectivity is an important strength of the manuscript, but its methodological description should be made more precise. In particular, the authors should clearly define the populations represented by the terms “baseline sample”, “survivors”, “invited participants”, and “follow-up sample”. This is especially important because some individuals were alive but had emigrated, were not residing in Sweden, or could not be contacted.

Similar clarification is needed for the mortality-associated and experimental components. The abbreviation “SMD” should preferably be used throughout for “standardised mean difference”, since “SD” conventionally refers to the standard deviation.

The manuscript also states that effect sizes for dichotomous variables were supplemented with rate ratios. However, these ratios do not appear to be reported in the supplementary tables. The authors should provide the corresponding estimates, describe their calculation, and specify the reference category for each variable. Confidence intervals would also improve the interpretation of the magnitude and precision of the reported estimates.

Interpretation of the response rate

The statement that the response rate was above a “critical threshold” of 30%, which has been found to substantially reduce non-response bias, should be reconsidered. Non-response bias cannot generally be inferred from the response rate alone, as it depends on the association between response propensity and the variables of interest. Indeed, the authors’ own analyses show non-negligible selectivity related to childhood cognitive ability and educational level.

I suggest removing the notion of a generally reassuring threshold and relying instead on the empirical selectivity analyses, which provide a much more informative assessment of the composition of the follow-up sample.

Comparability of cognitive assessments across the lifespan

One of the most distinctive features of UGU-LIFE is the administration in later life of cognitive tests originally completed at age 13. However, the assessment conditions differed substantially across waves. The childhood tests were completed on paper, at school, and under teacher supervision, whereas the follow-up tests were administered online and apparently without direct supervision. Five additional items were also added to the verbal knowledge test.

These differences may affect direct comparisons between childhood and late-adulthood performance. Accordingly, statements suggesting that the repeated measures allow a straightforward assessment of long-term cognitive change should be qualified.

Moreover, the manuscript mentions a pilot study in which half of the participants completed paper-and-pencil versions of the cognitive tests specifically to examine mode effects. However, no results from this comparison are presented. Given the centrality of longitudinal cognitive comparisons to the aims of the cohort, the authors should report at least the main findings of the mode-effect analysis. If adequate evidence of comparability is not yet available, this should be explicitly acknowledged as a limitation.

Documentation and psychometric evaluation of the learning tasks

The food-ordering and vocabulary-learning tasks are central to the stated purpose of UGU-LIFE. Nevertheless, the current description provides limited information about their psychometric properties and data-processing procedures.

The manuscript would benefit from reporting basic descriptive information on task performance, including score distributions, floor or ceiling effects, and preliminary reliability or validity evidence where available. The authors should also explain more clearly how partially completed tasks were scored and how valid task completion was defined.

Relatedly, Table 6 reports different exclusion thresholds for missing data, including more than 85%, 86%, or 98% missing values. The rationale for these task-specific thresholds is not evident. The authors should describe and justify the data-cleaning rules and clarify whether these rules were established before the present analyses.

Consent and ethical procedures

Although the manuscript reports approval from the Swedish Ethical Review Authority, the procedures for obtaining consent require further description. The authors should clarify how consent was obtained for:

- participation in the follow-up survey and cognitive or learning assessments;
- saliva collection and genetic analysis;
- linkage with socioeconomic and health registries;
- continued use of data from individuals who were deceased or could not be contacted.

The opt-out procedure for registry linkage should also be explained more fully, including how participants were informed and how the procedure applies to individuals from the original cohorts who did not participate in the new assessment.

Content of the “Findings to date” section

The “Findings to date” section is currently focused almost entirely on sample retention and selectivity. These analyses are useful, but the inclusion of at least some descriptive findings from the cognitive and learning assessments would make the cohort profile more

informative. In particular, summary statistics for the main cognitive tests and learning outcomes would help readers understand the nature, range, and quality of the newly collected data.

If the authors consider these results premature, this should be stated explicitly, and the section could be reframed as describing cohort participation and data quality rather than substantive findings.

Minor comments

Table 6 includes columns for both frequencies and percentages, but percentages are missing for most cognitive tests, learning tasks, and DNA sampling. These should be completed.

The final page of Supplementary Table 7 is numbered “Page 42 of 41”. The pagination of the supplementary material should be corrected.

The description of the metal-folding task is difficult to understand and should be rewritten. In particular, the sentence describing how participants identify the figure resulting from folding the presented pattern is grammatically unclear.

The distinction between the representativeness of the original cohorts and that of the follow-up sample should be maintained consistently. The original samples were nationally representative, whereas the follow-up sample shows substantial selectivity related to education and childhood cognitive ability.

As DNA collection was still ongoing at the time covered by the manuscript, the reported number of samples should be explicitly described as provisional. The authors should also distinguish between samples received, DNA successfully extracted, samples genotyped, and samples passing genotyping quality control.

The wording of some sentences would benefit from language editing. Examples include “the sampling frame was estimated to” and several descriptions of the cognitive tasks.

The meaning of “task completion” should be stated consistently. At present, participants are included in the follow-up sample if they completed at least one of seven components, but some components were partially completed or subsequently subjected to data-cleaning exclusions.

VERSION 1 - AUTHOR RESPONSE

Reviewer: 1
Ms. Cao Huizhen, Fuzhou University Affiliated Provincial Hospital
Comments to the Author:
This paper aims to established to investigate the conditions shaping lifelong learning. The cohort reframing aging as a time of active knowledge production and inclusive lifelong learning systems and age-friendly societies. The greater impact of lifelong learning and selective attrition of the order people can be associated with activities of daily living, vision and hearing. Details of the cohort how to evaluate these factors are, however, not clear.

RESPONSE: We agree that activities of daily living, vision and hearing are relevant factors in this context. Functional limitations at follow-up are measured as part of the Short Form Health survey instrument (SF-36) and medical records (of both inpatient and outpatient care) are collected from the National Patient Register (see Table 2). For the purpose of this paper, we assess health-related selectivity at follow-up with the Charlson Comorbidity Index (see p. 16, Table 3 and 4, and Figure 4 and 5).

Reviewer: 2
Dr. Marcus Richards, University College London
Comments to the Author:
Education though out life is an important yet a relatively under-studied topic. This is a well-written and comprehensive description of the UGU-LIFE study. However, I would suggest three minor revisions.

First, re the Strengths subsection, while the simultaneous availability of cognition in childhood and adulthood is rare, it is not - as claimed by the authors - unique. For example, both are available in the British 1946 birth cohort and follow-ups of the Scottish Mental Surveys.

RESPONSE: We agree and have removed the implication that the availability of childhood and adult cognitive assessments is unique to UGU-LIFE. We now describe this as a rare and valuable feature and acknowledge the British 1946 birth cohort and the Lothian Birth Cohorts, which follow participants from the Scottish Mental Surveys (see "Strengths and limitations of this study" on p. 3 and "Strengths and limitations" on p. 20). We have added the cohort profile references by Wadsworth et al. and Deary et al.

Second, while the authors comment on the low retention rate for follow-up (26.2%), this is in fact a serious limitation.

RESPONSE: We agree that the original discussion did not adequately convey the seriousness of this limitation. We have strengthened this acknowledgment both in the summary and the main limitations section (see "Strengths and limitations of this study" on p. 3 and "Strengths and limitations" on p. 21).

Third, the authors should comment on the risk of consultation bias in the use of health registry data.

RESPONSE: We agree that consultation bias is an important limitation of registry-based health information. We have therefore clarified in the description of the National Patient Register that recorded diagnoses depend on healthcare contact and clinical recording, and that absence of a registered diagnosis should not be interpreted as absence of the underlying condition (p. 14). We have also added a paragraph to the Strengths and limitations section noting that registry-based morbidity may be influenced by healthcare-seeking behaviour, access to care, referral patterns, and diagnostic practices (p. 21).

Reviewer: 3

Dr. Alessandro Oronzo Caffo, Universita degli Studi di Bari Aldo Moro

Comments to the Author:

This manuscript presents the cohort profile of the Swedish Evaluation Through Follow-up Study of Learning Later in Life (UGU-LIFE), which builds on two nationally representative Swedish birth cohorts originally assessed at age 13 and followed up approximately six decades later. The study combines childhood survey and cognitive data with cognitive testing, direct learning tasks, psychosocial measures, nationwide registry data, and genetic sampling in later life. Overall, this is a valuable and potentially important research resource. The exceptionally long follow-up period, the repeated administration of cognitive measures, and the integration of multiple data sources provide considerable opportunities to investigate learning and cognitive functioning from a lifespan perspective. The manuscript is generally well organised, and the explicit examination of mortality-related and experimental selectivity is particularly welcome. I believe that the cohort profile is suitable for publication. However, several aspects of the reporting require clarification or correction before the manuscript can provide a sufficiently precise and reproducible description of the cohort. I therefore recommend major revision, although the issues identified below appear to be addressable without fundamental changes to the study.

Major comments

Inconsistency in the distribution of sex at follow-up

There is a clear inconsistency between the overall and cohort-specific figures reported in Table 3. In the overall follow-up sample, the table reports 3,034 men and 2,704 women. However, the cohort-specific figures sum to 2,704 men (1,406 + 1,298) and 3,034 women (1,476 + 1,558). The cohort-specific percentages also indicate that women represented approximately 51% of the 1948 cohort and 55% of the 1953 cohort at follow-up.

The same apparent inversion is reproduced in the main text, where the authors state that the follow-up sample included 3,034 men and 2,704 women. The authors should correct these figures and verify whether the error is confined to the presentation of the descriptive data or also affects the sex-related selectivity analyses and the corresponding figures.

> **RESPONSE:** Thank you for identifying this error. Indeed, the sex distributions of the overall follow-up sample were mistakenly misplaced while the cohort-specific numbers are correct. The follow-up sample includes 3,034 women (1,476 + 1,558) and 2,704 men (1,406 + 1,298). These figures (Table 3 and p. 15 in main text) have been corrected in the revised manuscript. We further confirm that the error only appeared in the presentation of descriptive data and did not affect the selectivity analyses and corresponding figures.

Definition and calculation of selectivity indicators

The analysis of selectivity is an important strength of the manuscript, but its methodological description should be made more precise. In particular, the authors should clearly define the populations represented by the terms “baseline sample”, “survivors”, “invited participants”, and “follow-up sample”. This is especially important because some individuals were alive but had emigrated, were not residing in Sweden, or could not be contacted. Similar clarification is needed for the mortality-associated and experimental components. The abbreviation “SMD”

should preferably be used throughout for "standardised mean difference", since "SD" conventionally refers to the standard deviation.

The manuscript also states that effect sizes for dichotomous variables were supplemented with rate ratios. However, these ratios do not appear to be reported in the supplementary tables. The authors should provide the corresponding estimates, describe their calculation, and specify the reference category for each variable. Confidence intervals would also improve the interpretation of the magnitude and precision of the reported estimates.

RESPONSE: We appreciate the need to enhance clarity in definition and calculation of selectivity indicators. In the revised manuscript, we have:

1. Defined the populations represented by the terms "baseline sample", "survivors", "invited participants", and "follow-up sample" (see p. 15-16).
2. Replaced "SD" with "SMD" when referring to standardized mean differences in the selectivity analyses (see Figure 4, Figure 5, and in-text effect size descriptions on p. 15–20).
3. Clarified how the rate ratios are calculated and that they are expressed as percentage differences in text (e.g., "Men were 10% less likely than women to be alive at follow-up"; see p. 16).
4. Added confidence intervals to all SMD estimates in Table 5 and 7.

Interpretation of the response rate

The statement that the response rate was above a "critical threshold" of 30%, which has been found to substantially reduce non-response bias, should be reconsidered. Non-response bias cannot generally be inferred from the response rate alone, as it depends on the association between response propensity and the variables of interest. Indeed, the authors' own analyses show non-negligible selectivity related to childhood cognitive ability and educational level.
I suggest removing the notion of a generally reassuring threshold and relying instead on the empirical selectivity analyses, which provide a much more informative assessment of the composition of the follow-up sample.

RESPONSE: We agree and have removed this paragraph from the manuscript (p. 8).

Comparability of cognitive assessments across the lifespan

One of the most distinctive features of UGU-LIFE is the administration in later life of cognitive tests originally completed at age 13. However, the assessment conditions differed substantially across waves. The childhood tests were completed on paper, at school, and under teacher supervision, whereas the follow-up tests were administered online and apparently without direct supervision. Five additional items were also added to the verbal knowledge test.
These differences may affect direct comparisons between childhood and late-adulthood performance. Accordingly, statements suggesting that the repeated measures allow a straightforward assessment of long-term cognitive change should be qualified.
Moreover, the manuscript mentions a pilot study in which half of the participants completed paper-and-pencil versions of the cognitive tests specifically to examine mode effects. However, no results from this comparison are presented. Given the centrality of longitudinal cognitive comparisons to the aims of the cohort, the authors should report at least the main findings of

the mode-effect analysis. If adequate evidence of comparability is not yet available, this should be explicitly acknowledged as a limitation.

> **RESPONSE:** Thank you for highlighting this important issue. We agree that the repeated administration of the same cognitive tests does not, by itself, imply full measurement comparability across childhood and late adulthood. Although the core content of the opposites, number series, and metal folding tests was retained, the assessment conditions differed across waves. We have therefore revised the manuscript to avoid wording that could be interpreted as implying a straightforward assessment of absolute cognitive change across the lifespan. We now explicitly state that differences in administration mode and testing context may affect cross-wave comparisons and should be taken into account when interpreting longitudinal change (p. 12).
>
> A detailed evaluation of mode effects, score comparability, and the psychometric properties of the cognitive measures is being conducted in a separate validation paper. These analyses require psychometric evaluation that goes beyond the descriptive purpose of the present cohort profile and, in our view, warrants dedicated treatment rather than a brief summary that might be difficult to interpret without the full psychometric context. Because that validation work is not yet published, we have not used it to claim equivalence between assessment modes in the present manuscript. Instead, we have revised the cohort profile to make clear that comparability across waves has not yet been fully established and that this represents an important consideration for future analyses of cognitive change (p. 20). We have also clarified that detailed findings regarding mode comparability will be reported separately (p. 14-15).

Documentation and psychometric evaluation of the learning tasks
The food-ordering and vocabulary-learning tasks are central to the stated purpose of UGU-LIFE. Nevertheless, the current description provides limited information about their psychometric properties and data-processing procedures.
The manuscript would benefit from reporting basic descriptive information on task performance, including score distributions, floor or ceiling effects, and preliminary reliability or validity evidence where available. The authors should also explain more clearly how partially completed tasks were scored and how valid task completion was defined.
Relatedly, Table 6 reports different exclusion thresholds for missing data, including more than 85%, 86%, or 98% missing values. The rationale for these task-specific thresholds is not evident. The authors should describe and justify the data-cleaning rules and clarify whether these rules were established before the present analyses.

> **RESPONSE:** We agree that the psychometric properties and scoring characteristics of the learning tasks require careful evaluation. We have chosen not to provide a brief or partial psychometric evaluation in the present cohort profile because these analyses require detailed consideration of trial-level performance, scoring decisions, and task-specific psychometric properties that warrant dedicated treatment in a separate validation paper. We have therefore revised the manuscript to make clear that the psychometric evaluation of the learning tasks is ongoing and that detailed validation results will be reported separately (p. 13).

We have further clarified the purpose of task-specific data processing in Table 6. The exclusions reported there were designed primarily to identify records containing sufficient data to indicate meaningful task participation for the present cohort-description and selectivity analyses. They should not be interpreted as general psychometric thresholds defining valid learning scores for future substantive analyses. Because the components differ in length and structure, the minimum amount of observed information needed to distinguish meaningful participation from records that had effectively not been completed results in different percentages of allowable missingness. Notably, there was a typo in the percentage reported for number series. For all cognitive tests, the minimum amount of observed information needed to distinguish meaningful participation was set to 15% (i.e., records with >85% missing were excluded). This has been corrected in the revised file.

Consent and ethical procedures

Although the manuscript reports approval from the Swedish Ethical Review Authority, the procedures for obtaining consent require further description. The authors should clarify how consent was obtained for:

– participation in the follow-up survey and cognitive or learning assessments;
– saliva collection and genetic analysis;
– linkage with socioeconomic and health registries;
– continued use of data from individuals who were deceased or could not be contacted.

The opt-out procedure for registry linkage should also be explained more fully, including how participants were informed and how the procedure applies to individuals from the original cohorts who did not participate in the new assessment.

RESPONSE: We have revised the cohort description to specify the consent procedures for the follow-up survey, cognitive and learning assessments, saliva collection and genetic analysis, and registry linkage (p. 7). The revised text also clarifies that registry linkage was approved for the full original UGU cohorts, including deceased and non-participating individuals, and that all living invitees were given the opportunity to opt out of registry linkage.

Content of the "Findings to date" section

The "Findings to date" section is currently focused almost entirely on sample retention and selectivity. These analyses are useful, but the inclusion of at least some descriptive findings from the cognitive and learning assessments would make the cohort profile more informative. In particular, summary statistics for the main cognitive tests and learning outcomes would help readers understand the nature, range, and quality of the newly collected data.

If the authors consider these results premature, this should be stated explicitly, and the section could be reframed as describing cohort participation and data quality rather than substantive findings.

RESPONSE: We agree that descriptive findings from the cognitive tests and learning tasks would be informative, but as mentioned above, these data are currently being processed and do not fit within the scope of the cohort profile. As suggested, we now explicitly state that these data are under evaluation (p. 13, 14-15) and that the

aim of the “findings to date” section is to report participation and selectivity at follow-up (p. 15).

Minor comments

Table 6 includes columns for both frequencies and percentages, but percentages are missing for most cognitive tests, learning tasks, and DNA sampling. These should be completed.

RESPONSE: The percentages were intended to describe subsample distributions (i.e., proportion of web vs. paper survey responses). We realize, however, that they may be informative also for describing the proportion of participants completing each task. We have therefore completed the figures as suggested (see Table 6).

The final page of Supplementary Table 7 is numbered “Page 42 of 41”. The pagination of the supplementary material should be corrected.

RESPONSE: The pagination “Page 42 of 41” is generated by the submission system and therefore beyond our control. The page numbers in the submitted documents (manuscript and supplementary material) have been checked for accuracy.

The description of the metal-folding task is difficult to understand and should be rewritten. In particular, the sentence describing how participants identify the figure resulting from folding the presented pattern is grammatically unclear.

RESPONSE: We have rewritten this sentence: "The metal folding test consisted of 40 items in which participants viewed a two-dimensional pattern and selected, from four alternatives, the three-dimensional figure that would result if the pattern were folded." We have also revised the description of the other cognitive tests to enhance language and clarity (p. 8-9).

The distinction between the representativeness of the original cohorts and that of the follow-up sample should be maintained consistently. The original samples were nationally representative, whereas the follow-up sample shows substantial selectivity related to education and childhood cognitive ability.

RESPONSE: We agree and have clarified limitations regarding representativity at follow-up on p. 3 and 21.

As DNA collection was still ongoing at the time covered by the manuscript, the reported number of samples should be explicitly described as provisional. The authors should also distinguish between samples received, DNA successfully extracted, samples genotyped, and samples passing genotyping quality control.

RESPONSE: Saliva samples are currently being processed for DNA extraction and genotyping at the Karolinska Institutet Biobank. The number of samples that are

successfully extracted and genotyped are therefore not yet known. The reported figures are thus restricted to the number of saliva samples collected between the 27th of June 2025 and the 12th of May 2026 (see p. 7, 18, and Table 6).

The wording of some sentences would benefit from language editing. Examples include "the sampling frame was estimated to" and several descriptions of the cognitive tasks.

RESPONSE: We have carefully reviewed the manuscript for language and clarity and revised grammatically unclear formulations. In particular, we changed "the sampling frame was estimated to" to "the sampling frame was estimated at," rewrote the descriptions of the cognitive tests, and edited wording throughout the cohort description, follow-up procedures, pilot-study description, findings to date section, figure captions, and data-sharing sections. These revisions were made to improve grammatical accuracy, readability, and consistency of terminology throughout the manuscript.

The meaning of "task completion" should be stated consistently. At present, participants are included in the follow-up sample if they completed at least one of seven components, but some components were partially completed or subsequently subjected to data-cleaning exclusions.

RESPONSE: We have clarified the meaning of task completion and how participation at follow-up is defined on p. 7-8.
